# IL17-deficient NOD mice are protected from autoimmune diabetes due to decreased antigen presentation and T cell activation

**DOI:** 10.3389/fimmu.2025.1728313

**Published:** 2026-01-12

**Authors:** James A. Pearson, Yangyang Li, Juan Huang, Jian Peng, F. Susan Wong, Li Wen

**Affiliations:** 1 Section of Endocrinology, School of Medicine, Yale University, New Haven, CT, United States; 2 Diabetes Research Group, Division of Infection and Immunity, School of Medicine, Cardiff University, Wales, United Kingdom; 3 Department of Endocrinology, Sir Run Run Hospital, Nanjing Medical University, Nanjing, Jiangsu, China

**Keywords:** gut microbiota, IL17, ILC, T cell, type 1 diabetes

## Abstract

**Introduction:**

IL-17 is a key cytokine helping preserve the intestinal barrier against infections; however, the T cells that primarily secrete IL-17 (Th17) can promote the development of autoimmunity. In Type 1 diabetes, the role of IL-17 is less well understood, with many studies evaluating the role of IL-17, without considering changes within the intestine. Furthermore, therapeutically targeting IL-12/IL-23 (upstream of IL-17) or IL-17 directly can help preserve insulin-producing beta cells in those newly diagnosed with Type 1 diabetes. Thus, there is a need to better understand how IL-17 may modulate susceptibility to Type 1 diabetes by linking intestinal changes to type 1 diabetes development.

**Methods:**

We studied IL-17-deficient NOD mice to understand the role of IL-17 in mediatingsusceptibility to Type 1 diabetes in vivo and in vitro.

**Results:**

Our study showed that IL-17-deficient NOD mice were protected from autoimmune diabetes, and *in vivo* adoptive transfer studies showed that both immune and non-immune cells are important for modulating diabetes development. We found significant reductions in both regulatory T cells and inflammatory T-bet-expressing CD8+ T cells, while Type 3 Innate Lymphoid Cells (ILC3s) were expanded. These changes were found to be mediated through altered gut microbiota composition of the IL-17-deficient NOD mice. Finally, we demonstrated that intestinal epithelial cells from IL-17-deficient NOD mice were less able to present autoantigen to autoreactive CD8+ T cells, with reduced proinflammatory cytokine secretion. This effect was specific to IL-17 deficiency, as addition of exogenous IL-17 resulted in improved antigen presentation to autoreactive CD8+ T cells.

**Discussion:**

Together, our data suggest a novel role for IL-17 in modulating epithelial cell function and antigen presentation within the intestinal tissue, resulting in reduced autoantigen-specific T cell responses and enhanced protection from autoimmune diabetes. Better understanding of how targeted IL-17 blockade could be administered to the intestine may help better prevent the development of Type 1 diabetes.

## Introduction

Intestinal microbiota composition, and the immune responses invoked against the microbiome, have been linked to susceptibility to cancer, infection and autoimmunity, including Type 1 diabetes. It is clear, in the context of Type 1 diabetes, that the gut microbiota composition (1–5), intestinal barrier permeability (6), and small intestinal morphology (7), are changed prior to disease development. Furthermore, fecal microbiota transplantation in those living with Type 1 diabetes has been shown to preserve the residual insulin-secreting beta cell function (8). Thus, interactions between the microbiota and immune system are important in contributing to susceptibility to Type 1 diabetes but many interactions still remain understudied.

A number of approaches have been utilized to improve intestinal homeostasis and barrier function including oral supplementation with short chain fatty acids, which held promise in the Non-obese diabetic (NOD) mouse model for spontaneous autoimmune diabetes (9, 10); however, their impact in humans with Type 1 diabetes has been variable (11, 12). Thus, more studies and new approaches are needed.

One approach that has been clinically successful in preserving beta cell function is to target either IL-12/IL-23 or IL-17 using monoclonal antibodies in those with newly diagnosed Type 1 diabetes (13, 14). These approaches are believed to work through reducing proinflammatory IL-17-secreting T cell populations (13), as IL-23 can promote IL-17 secretion from T cells (15).

IL-17A was the first of six IL-17 family members, cloned from a T cell hybridoma (16), which can bind to the IL-17 receptor found in many different tissues across the body (17). T cells are the primary source of IL-17A, secreted mainly by a specialized subset of CD4^+^ T cells named Th17 T cells (15, 18, 19), and aids in the protection of mucosal barriers e.g. intestine, against microorganisms such as bacteria, viruses and fungi. In addition to Th17 cells, many other immune cells including T-bet^+^CD8^+^ T cells (20), γδ T cells (21) and innate lymphoid cells (22), as well as non-immune cells such as intestinal epithelial cells and Paneth cells (23, 24) secrete IL-17A.

Th17 T cells are involved in mediating the pathogenesis of multiple autoimmune diseases including multiple sclerosis and rheumatoid arthritis (25, 26); however, the role of IL-17 in Type 1 diabetes has not been consistent. In Non-obese diabetic (NOD) mice, which develop spontaneous autoimmune diabetes, IL-17 gene silencing (using lentiviruses introduced into NOD zygotes) showed no protection from Type 1 diabetes (27). Similarly, treatment with a neutralizing anti-IL-17 antibody in young (<5 week old) NOD mice also had no effect on diabetes development; however, the same treatment in 10-week old NOD mice significantly reduced diabetes development through Treg expansion (28). This variability in response to blockade of IL-17 may be linked to changes in gut microbiota composition as it is well known that bacteria such as Segmented Filamentous bacteria (SFB) can induce Th17 cells within the intestine (29) and that SFB colonization in female NOD mice, but not male NOD mice, is linked to protection from Type 1 diabetes (30). No studies have investigated how IL-17 deficiency has a combined effect on gut microbiota and diabetes susceptibility. Thus, we investigated whether IL-17 gene-deficient NOD mice exhibited altered gut bacterial composition and whether that resulted in differences in susceptibility to Type 1 diabetes.

## Materials and methods

### Mice

NOD/Caj mice have been maintained at Yale University for 30 years. IL-17^-/-^NOD mice were generated by backcrossing IL-17^-/-^C57BL/6 mice (kindly provided by Dr. Richard Flavell, Yale University) to the NOD background for over 10 generations. IL-17^-/-^NOD mice were then crossed to both NOD.scid and BDC2.5 T cell receptor transgenic mice. The purity of the NOD genetic background was verified by Illumina mouse whole genome SNP scan (DartMouse™). BDC2.5 and NY8.3 T cell receptor transgenic NOD mice, as well as NOD.scid mice, were originally obtained from the Jackson Laboratory and have been bred in-house for about 3 decades. Mice of both sexes were studied. All the mice were housed in individually-ventilated filter cages (IVC) in specific pathogen-free (SPF) conditions in a 12-hour dark/light cycle and received autoclaved food ad libitum. The Institutional Animal Care and Use Committee at Yale University approved the use of mice and the procedures in this study. All mice were euthanized by cervical dislocation.

### Diabetes monitoring

Mice were monitored for glycosuria weekly, from 12-weeks of age, for spontaneous diabetes development, with diabetes confirmed the next day by blood glucose (>250mg/dl/13.9mmol/l).

### Histology

Pancreata from 12-week-old female mice were collected and formalin-fixed. Sections from 2–3 different layers within each pancreata were cut to 8μM thick and hemotoxylin and eosin stained (Department of Pathology, Yale). Individual islets were scored under light microscopy by a blinded scorer for no insulitis; less than <25% infiltration of the islet; 25–50% infiltration; 50-75% infiltration and >75% infiltration. A range of 217–243 islets were scored for insulitis in each group (n = 4 mice). Significance was determined using a Chi square test. **p < 0.01.

### Cell staining and flow cytometry

Single cell suspensions were prepared from splenocytes, pancreatic lymph nodes (PLNs), mesenteric lymph nodes (MLNs) and Peyers’ patches (PP), harvested from 6-8-week-old mice. Cells were incubated with Fc block (2.4G2) for 10 mins at room temperature prior to mAb staining and a cell viability dye (Zombie Aqua Fixable viability dye, BioLegend) for 30 mins at 4°C. All the antibodies used in this study were from Biolegend and diluted 1:400 to 1:500 unless otherwise stated. Cells were stained with combinations of mAbs to TCRβ (H57-597), CD4 (GK1.5), CD8α (53-6.7), CD25 (PC61), CD44 (IM7), CD62L (MEL-14), TCRγδ (GL3), CD19 (6D5), B220 (RA3-6B2), MHCII (10-3.6), CD11b (M1/70), CD11c (N418). Cells were then washed and kept at 4°C until analysis. For transcription factor staining, cells were stained with mAbs to the surface markers as above, prior to fixation (60 mins at room temperature in the dark) and permeabilization, using Foxp3/Transcription Factor Staining Buffer Set (Tonbo Biosciences). Cells were stained intracellularly with mAbs to RORγt (Q31-378, BD), T-bet (4B10), GATA-3 (16E10A23) and FoxP3 (FJK-16s, eBioscience). Samples were analyzed on a BD LSRFortessa Flow Cytometer and results were analyzed by FlowJo v8.8.6 and 10.4 (Treestar/BD). Fluorescent minus one (FMO) controls and isotype controls were used to determine appropriate gating.

### Cell isolation and enrichment

Splenic T cells were purified from 6-8-week-old donor mice using hybridoma supernatants containing mAbs to either CD4 (GK1.5) or CD8 (TIB105) and I-A^g7^ (10.2.16, recognizing MHC class II I-A^g7^ of the NOD mouse), generously provided by the late Charles Janeway Jr. (Yale University) for 30 mins at 4°C. Cells were then washed in PBS and incubated for 45 mins on ice with magnetic beads conjugated with goat anti-mouse IgG, goat anti-mouse IgM or goat anti-rat IgG (QIAGEN). T cells were magnetically isolated using negative selection. The purity of the T cells was 95-99%, as verified by flow cytometry.

### *In vivo* adoptive transfers

10^7^ splenocytes from recently diagnosed diabetic IL-17-sufficient or -deficient mice were intravenously transferred into NOD.scid mice. 4-week-old IL-17-sufficient or -deficient NOD mice were irradiated (600 rads) for use as recipients of 10^7^ splenocytes from recently-diagnosed diabetic IL-17-sufficient mice. Splenocytes were harvested from 6-week-old IL-17-sufficient or -deficient BDC2.5 T cell receptor transgenic NOD mice and co-cultured *in vitro* for 2 days in the presence of 10ng/ml BDC2.5 mimotope (RTRPLWVRME), prior to CD4^+^ T cell isolation by magnetic isolation and subsequent adoptive transfer of 3x10^6^ BDC2.5 CD4^+^ T cells into IL-17-sufficient or -deficient NOD.scid recipients. Recipients were screened for diabetes development by testing for glycosuria daily, with mice terminated upon diabetes development, confirmed by blood glucose >13.9mmol/l or after 80 days post-transfer.

### Bacterial DNA extraction and analysis

Fecal samples from 8-week-old mice were collected and stored at −20°C. Bacterial DNA was extracted as previously described (31). In brief, the fecal samples were resuspended in 300 µl TE buffer (10 mM Tris and 1 mM EDTA, pH 8) containing 7.5 µl SDS (0.5%) and 3 µl proteinase K (20 mg/ml) and incubated for 1 hour at 37°C (all Sigma-Aldrich). One volume of phenol/chloroform/isoamyl alcohol (25:24:1), 200 µl of 20% SDS, and 0.3 g of zirconium/silica beads (0.1 mm; BioSpec, Bartlesville, OK, USA) were added, and samples were mixed with a Mini bead-beater (BioSpec) for 2 min. The samples were then mixed with 820 µl phenol/chloroform/isoamyl alcohol (25:24:1) and centrifuged, and the aqueous layer was collected into a new tube. The bacterial DNA was precipitated with 0.6 volume of isopropanol, washed with 70% ethanol, air dried, and resuspended in 100 µl of sterile water. The V4 region of the bacterial 16S ribosomal gene was amplified from each DNA sample with barcoded broadly conserved bacterial primers (forward (5′-GTGCCAGCMGCCGCGGTAA-3′) and reverse primer (5′-GGACTACHVGGGTWTCTAAT-3′)). The PCR products were purified with a gel extraction kit (QIAGEN) and quantified with a spectrophotometer (Nanodrop), and equimolar amounts of each sample were pooled and pyrosequenced on an Ion Torrent personal genome machine sequencing system (Thermo Fisher Scientific). The results were analyzed using the QIIME software package (version 1.8) and UPARSE pipeline (version 7.0). After removing the primer sequences, the sequences were demultiplexed, quality filtered using QIIME, and further quality and chimera filtered in UPARSE pipeline. Operational taxonomic units (OTUs) were picked with 97% identity in UPARSE pipeline. In QIIME, the Greengenes reference database was used for taxonomy assignment, which was performed at various levels using representative sequences of each OTU. β-diversity was calculated to compare differences between microbial community profiles, and the data are shown as a principal coordinate analysis (PCoA). Microbial sequencing data can be accessed (NCBI submission ID SUB15501095 and BioProject ID: PRJNA1298311).

### Gavage of GF mice

Fresh fecal pellets from 6-8-week-old donor mice were resuspended in sterile PBS and homogenized using a Mini bead-beater (30secs; BioSpec). Fecal material was then centrifuged for 2 mins at low speed (52 xg) to remove dietary residue. The supernatant was transferred to a new tube and spun. After repeating the process twice, the combined supernatant was centrifuged at 469 xg to remove mammalian cells. Bacteria in the supernatant were pelleted by centrifugation at high speed (1876 xg, 5 min) and resuspended in PBS. Bacterial colony forming units (CFUs) were determined by measurement of optical density, which was pre-determined using an *E. coli* strain, using a spectrophotometer (Bio-Rad). Four-week-old GF NOD mice were colonized with 200 μl sterile PBS containing 2x10^8^ CFUs of stool bacteria by oral gavage. Colonized mice were terminated 2 weeks after gavage.

### qPCR

Intestinal tissues were stored in Trizol (Sigma-Aldrich) at -80°C, prior to RNA extraction and clean up using the RNeady Mini Kit (QIAGEN). Purified B cells were directly processed for RNA extraction. Equimolar concentrations of RNA were subsequently used for cDNA synthesis using an iScript™ cDNA Synthesis Kit (Bio-Rad, Hercules, CA, USA). qPCR was performed using an iQ5 qPCR detection system (Bio-Rad) according to the manufacturer’s instructions. The relative mRNA abundance was determined using the 2^−ΔΔCt^ method by normalization with the housekeeping gene GAPDH or 16S rRNA values (SFB). All primer sequences are listed in **Supplementary Table 1**.

### Cytokine ELISA

Cell culture supernatants and/or intestinal flush were assessed for IL17A (Mouse IL17A ELISA Ready-SET-GO! Kit, eBioscience), TGF-β1 and IL-18 (Mouse TGF-β1 or Mouse IL-18 DuoSet ELISA, Bio-techne), MIP-1β (Mouse CCL4/MIP-1β quantikine ELISA kit, Bio-techne) and IL-22, IL-23 (p19/p40) and IFNγ (ELISA MAX Deluxe Set, BioLegend) following the manufacturers’ protocols. The results were analyzed on a microplate spectrophotometer (Perkin Elmer), with cytokine concentrations determined by linear regression of each standard curve.

### Intestinal permeability assay

Mice were fasted overnight before gavage with FITC-dextran (600 mg/kg; ~4000 MW, Sigma-Aldrich). Two hours post-gavage, food supply was restored to the mice. After a further two hours, blood samples were collected from the mice and serum was isolated by centrifugation (2300 xg, 5 mins, room temperature). Serum samples were diluted 1:1 in PBS in a 96-well plate and FITC-dextran concentrations in the serum were determined using a fluorescence spectrophotometer (Perkin Elmer). Serum samples from non-FITC-dextran gavaged mice were used as baseline. Standard curves were generated using known concentrations of FITC-dextran diluted in control serum. Concentrations were determined using linear regression of a standard curve.

### Intestinal epithelial cell co-culture

Intestinal epithelial cells were isolated from the cecum of 6-8-week-old donor mice. Cecal epithelial cells were used as they have improved viability over small intestinal epithelial cells and also to more closely reflect intestinal epithelial cells exposed to microbiota identified in the fecal microbiota sequencing. The cecum was placed into ice-cold HBSS and flushed with 10ml ice-cold HBSS. The tissue was cut longitudinally and washed thrice using fresh ice-cold HBSS and then further dissected into tissue 1–2 cm long. Tissue was then transferred into 15 mls of HBSS containing 10 mM HEPES, 10 mM EDTA, Gentamycin, 2% FBS and 100 g/ml DNase I and incubated for 15 mins in 37°C-warmed shaking incubator. Post-incubation, tissue was incubated on ice for 10 mins and then transferred to 15 mls of HBSS (as above, without EDTA) and vigorously shaken for 30 secs. Epithelial cells were filtered through a 100 μm cell strainer, then treated with mitomycin-c for 20 mins at 37°C and washed twice. Epithelial cells were co-cultured 1:1 with magnetically isolated NY8.3 CD8^+^ T cells in the presence/absence of IGRP_206–214_ peptide and exogenous IL-17A (Bio-techne) for 48 hours. Half of the volume of the culture supernatants was removed, followed by adding ^3^H-thymidine for the final 18 hours to assess T cell proliferation.

## Results

### IL-17-deficiency protects NOD mice from diabetes development

**Figure 1:**
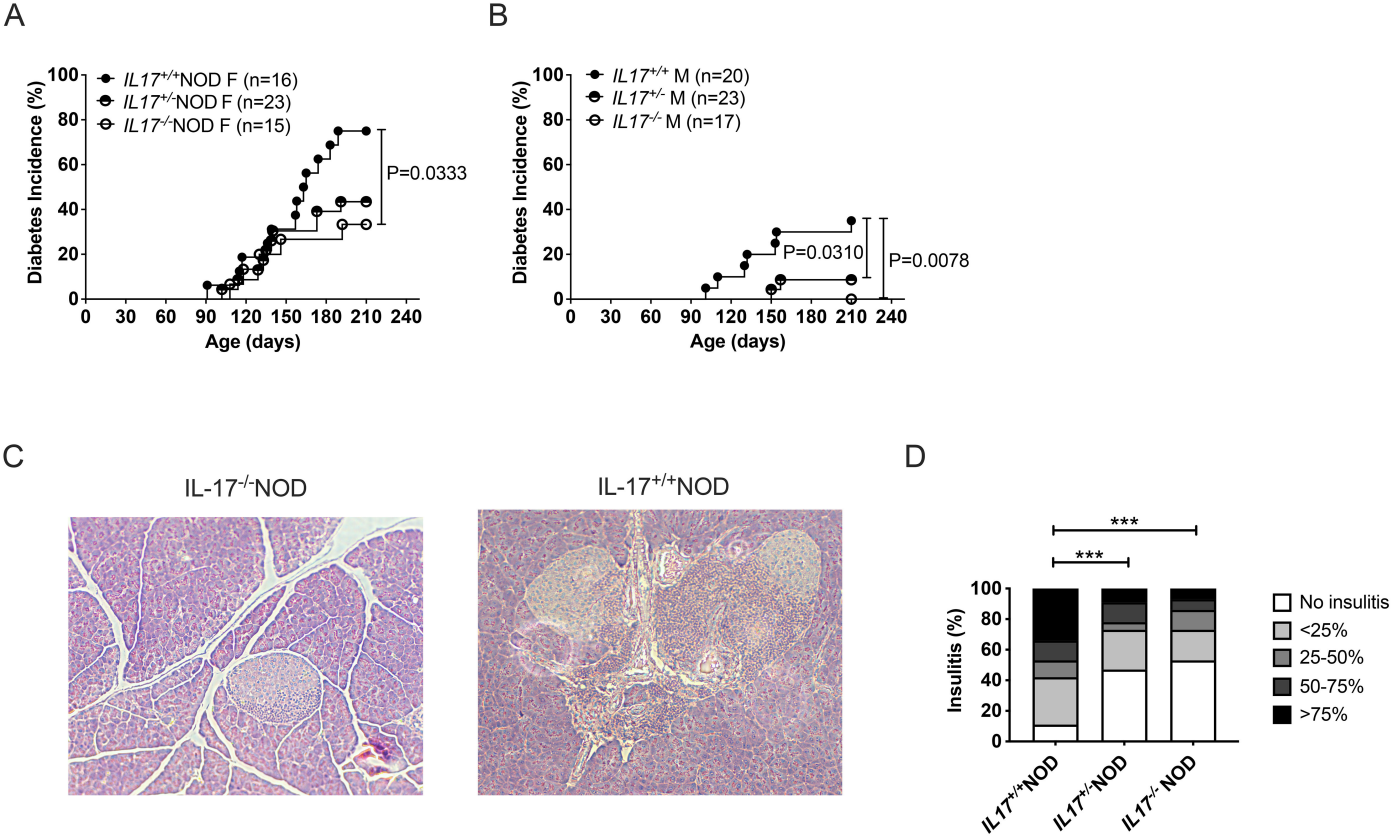
IL-17-deficiency protects NOD mice from development of Type 1 diabetes. Female **(A)** and male **(B)** NOD wild-type (IL-17^+/+^NOD), heterozygous (IL-17^+/-^NOD) or homozygous (IL-17^-/-^NOD) for IL-17 gene deficiency were studied for spontaneous diabetes development. **(C, D)** Immune infiltration of the pancreas was assessed from 12-week-old female NOD mice (n=4). Representative images of islet infiltration are shown **(C)** at 100x magnification and summarised using a five-grade scale **(D)** No insulitis, less than 25% immune infiltration, 25-50% infiltration, 50-75% infiltration and over 75% infiltration. Over 200 islets were counted per genotype. Data were assessed for significance using a log-rank (Mantel-Cox) test **(A, B)** or a Chi-square test **(D)**. p<0.0005.

To determine whether IL-17-deficiency influences susceptibility to Type 1 diabetes in NOD mice, we observed IL-17-sufficient, heterozygous and homozygous knock-out littermate mice for diabetes development. We found that mice lacking IL-17 were more protected from diabetes development than wild-type mice in both females and males (**Figures 1A, B**). Interestingly, even IL-17 heterozygous mice were protected from diabetes development compared to both female (p=0.07, **Figure 1A**) and male (p=0.03, **Figure 1B**) wild-type mice. In line with the diabetes incidence data, we found that there was less immune infiltration of the islets from 12-week-old IL-17-deficient and IL-17 heterozygous NOD mice, compared to wild-type IL-17-sufficient mice (**Figures 1C, D**).

### IL-17-deficiency modulates T cell and ILC3 proportions

**Figure 2:**
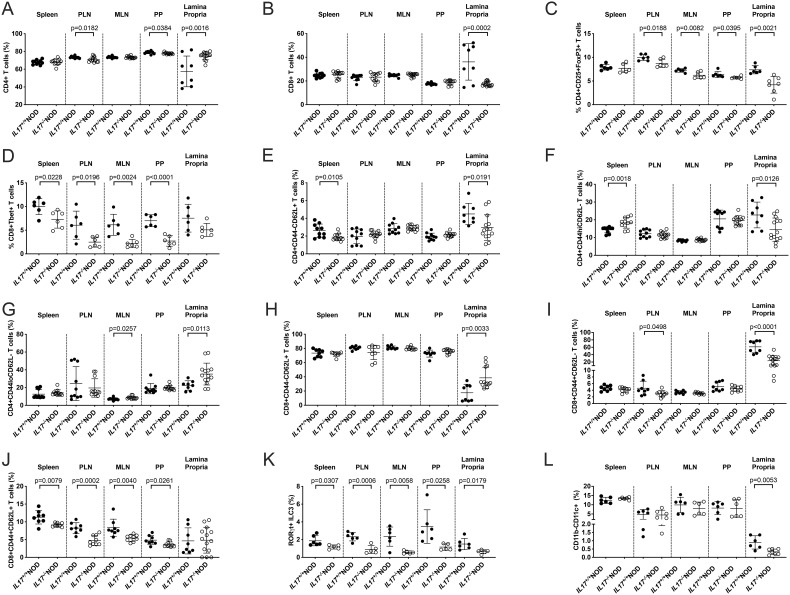
Immune cell proportion changes in IL-17-deficient NOD mice. Single cell suspensions were generated from 6-week-old mice (both sexes combined; n=6-8) from the spleen, pancreas-draining lymph node (PLN), mesenteric lymph node (MLN), Peyer’s Patches (PP) or small intestinal lamina Propria. T cells were gated from live single TCRβ^+^ cells prior to gating on CD4^+^**(A)** or CD8^+^**(B)** T cells. Tregs **(C)** were gated as A prior to subsequent gating on CD25^+^FoxP3^+^ cells, while T-bet^+^CD8^+^ T cells **(D)** were gated from **(B, F–K)** T cell activation was assessed using CD62L and CD44 expression for both CD4^+^ T cells **(F–H)** or CD8^+^ T cells **(I–K)**. **(L)** RORγt ILC3s were gated from live, single TCRβ^-^TCRγδ^-^RORγt^+^ cells, while CD11b^-^CD11c^+^ cells **(M)** were gated from live, single TCRβ^-^CD19^-^ cells. Data were assessed for significance by either a Student’s T-test (if normally distributed) or a Mann-Whitney test (if not normally distributed). Lines represent mean + SD. Data were pooled from 2 independent experiments.

As T cells can both secrete IL-17 and destroy the insulin-secreting beta cells to cause Type 1 diabetes, we asked whether there were changes in T cell phenotype that could explain the differences observed in diabetes development. We chose to study 6-8-week-old mice in the early stages of the autoimmune process to better understand the immune changes prior to diabetes development. We found modest reductions in the frequency of CD4^+^ T cells in both the pancreas-draining lymph nodes (PLNs) and Peyer’s patches in IL-17-deficient mice compared to their WT counterparts (**Figure 2A**). In contrast, we observed that CD4^+^ T cells were significantly increased in the lamina propria in IL-17 deficient mice, whereas CD8^+^ T cells were decreased (**Figures 2A, B**). To determine whether this difference was due to Th17 cells, which express the intranuclear transcription factor Rorγt (32), we examined CD4^+^Rorγt^+^ T cells. Unexpectedly, we found no significant difference in the proportion of Th17 CD4^+^ T cells, although there was a non-statistically significant trend to reduction (**Supplementary Figure S1A**). Similarly, we saw no significant difference in the proportion of CD8^+^Rorγt^+^ T cells (**Supplementary Figure S1B**). As T cells can be differentiated into Th17 cells, Tregs (FoxP3-expressing) and Th1 T cells (T-bet-expressing) and inter-convertible in different conditions, we examined whether there were any changes in these subsets. We found that Treg proportion was significantly reduced in most of the lymphoid tissue assessed in the IL-17-deficient mice (**Figure 1C**), while no significant differences were found in T-bet^+^CD4^+^ Th1 T cells (**Supplementary Figure S2C**). However, we observed a significant reduction in T-bet^+^CD8^+^ T cells in IL-17-deficient NOD mice (**Figure 1D**; **Supplementary Figure S1D**), which have been reported to aggressively attack islet beta cells (33). To ask whether IL-17-deficiency impacted T cell activation, we studied expression of CD44 and CD62L. We identified reduced naïve (CD44^-^CD62L^+^) but increased activated (CD44^hi^CD62L^-^) CD4^+^ T cells in splenocytes, whereas in the lamina propria, both naïve and activated CD4^+^ T cells were reduced while CD44^lo^CD62L^-^ T cells were increased (**Figures 2E–G**; **Supplementary Figure S1E**). Furthermore, we found expanded naïve but reduced effector (CD44^+^CD62L^-^) and central memory (CD44^+^CD62L^+^) CD8^+^ T cell subsets (**Figures 2H–J**; **Supplementary Figure S1F**). However, we observed no differences in the proportion of γδ T cells (**Supplementary Figure S1G**). This suggested that IL-17-deficiency impacted both CD4^+^ and CD8^+^ T cell subset frequencies, as well as activation, but surprisingly, the most significant differences were found in the CD8^+^ T cells.

As IL-17 is expressed by other cell types, including innate lymphoid cells (ILC3s), dendritic cells, macrophages and B cells, we investigated whether there were any changes in these subsets. We found that proportion of ILC3s (RORγt^+^) was reduced in all tissues assessed, while CD11c^+^CD11b^-^ dendritic cells were reduced only in the lamina propria and no differences were seen in CD11b^+^CD11c^-^ macrophages, or CD11b^+^CD11c^+^ cells or CD19^+^ B cells in IL17-deficient mice compared to IL-17-sufficient mice (**Figures 2K, L**; **Supplementary Figure S2**). Furthermore, no differences were seen in the proportion of other innate lymphoid cells (ILC1s (T-bet^+^) or ILC2s (GATA-3^+^)) (**Supplementary Figure S3**). Thus, IL-17-deficiency impacted the proportion and activation of T cells and ILC3s.

### IL-17-deficiency in both donors and recipients delays diabetes development

**Figure 3:**
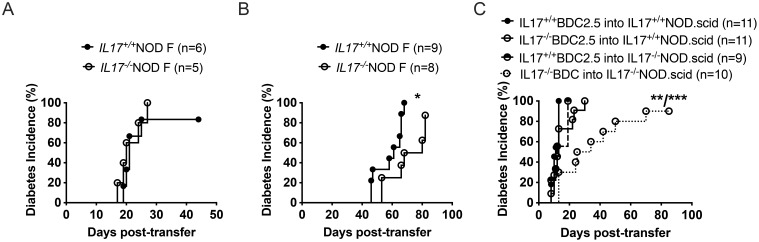
Immune and non-immune cells contribute to diabetes protection in adoptive transfer studies. **(A)** 10^7^ spleen cells from recently diagnosed diabetic IL-17^+/+^ or IL-17^-/-^NOD female mice were transferred into IL-17-sufficient NOD.scid mice. **(B)** 10^7^ spleen cells from recently diagnosed diabetic IL-17^+/+^NOD mice were transferred into irradiated young IL-17^+/+^ or IL-17^-/-^NOD mice. **(C)** 3x10^6^*in vitro* antigen-activated BDC2.5 CD4^+^ T cells (from IL-17^+/+^ or IL-17^-/-^ BDC2.5 NOD mice) were transferred into IL-17^+/+^ or IL-17^-/-^NOD.scid mice. Diabetes was diagnosed following a positive glycosuria test and a blood glucose >250mg/dl (13.9mmol/l). Data shown are pooled from two-three independent experiments and were assessed for significance using a Two-way ANOVA.

To determine whether changes observed in the immune cell phenotype were primarily responsible for protecting the IL-17-deficient mice from diabetes, we adoptively transferred spleen cells from recently diagnosed diabetic IL-17-sufficient and -deficient NOD mice into immunodeficient IL-17-sufficient NOD.scid mice. We found no significant difference in the ability of these spleen cells to transfer diabetes (**Figure 3A**). To ask whether this lack of difference in diabetes transfer was due to the host being IL-17-sufficient, we irradiated IL-17-sufficient or -deficient NOD mice and adoptively transferred spleen cells from diabetic IL-17-sufficient NOD mice. Here we found that IL-17-sufficient spleen cells transferred diabetes into IL-17-deficient mice but development of diabetes was delayed (**Figure 3B**). This suggested that IL-17-deficiency in the host was an important contributor to the development of type 1 diabetes. Finally, to determine the contribution between autoantigen-specific T cell IL-17 expression and host IL-17 expression, we generated two new mouse lines. One line involved breeding the BDC2.5 T cell receptor (TCR) transgenic NOD mice (34, 35) to the IL-17-deficient NOD mice (IL17^-/-^BDC2.5), allowing us to determine the contribution of autoantigen-specific CD4^+^ T cells in their ability to transfer diabetes. The second line involved breeding NOD.scid mice onto the IL-17-deficient NOD background to generate IL-17-deficient NOD.scid mice. Through a criss-cross experimental design, we were able to transfer either activated IL-17-sufficient BDC2.5 CD4^+^ T cells into IL-17-sufficient or -deficient NOD.scid mice. Similarly, we could also transfer IL-17-deficient BDC2.5 CD4^+^ T cells into IL-17-sufficient or -deficient NOD.scid mice. We found that only adoptive transfer of IL-17-deficient BDC2.5 CD4^+^ T cells into IL-17-deficient NOD.scid mice delayed the development of diabetes (**Figure 3C**). The results from this series of adoptive transfer experiments suggested that the protection observed in the IL-17-deficient NOD mice involves both immune and non-immune compartments.

### Gut microbiota from IL-17 deficient NOD mice partially transfers phenotype to germ free NOD mice

IL-17 is known to be produced by immune cells in response to the gut microbiota composition (29). Thus, to determine whether IL-17-deficiency and the protection from diabetes was related to gut microbiota changes, we first determined the composition of the microbiota between IL-17-sufficient and -deficient NOD mice.

**Figure 4:**
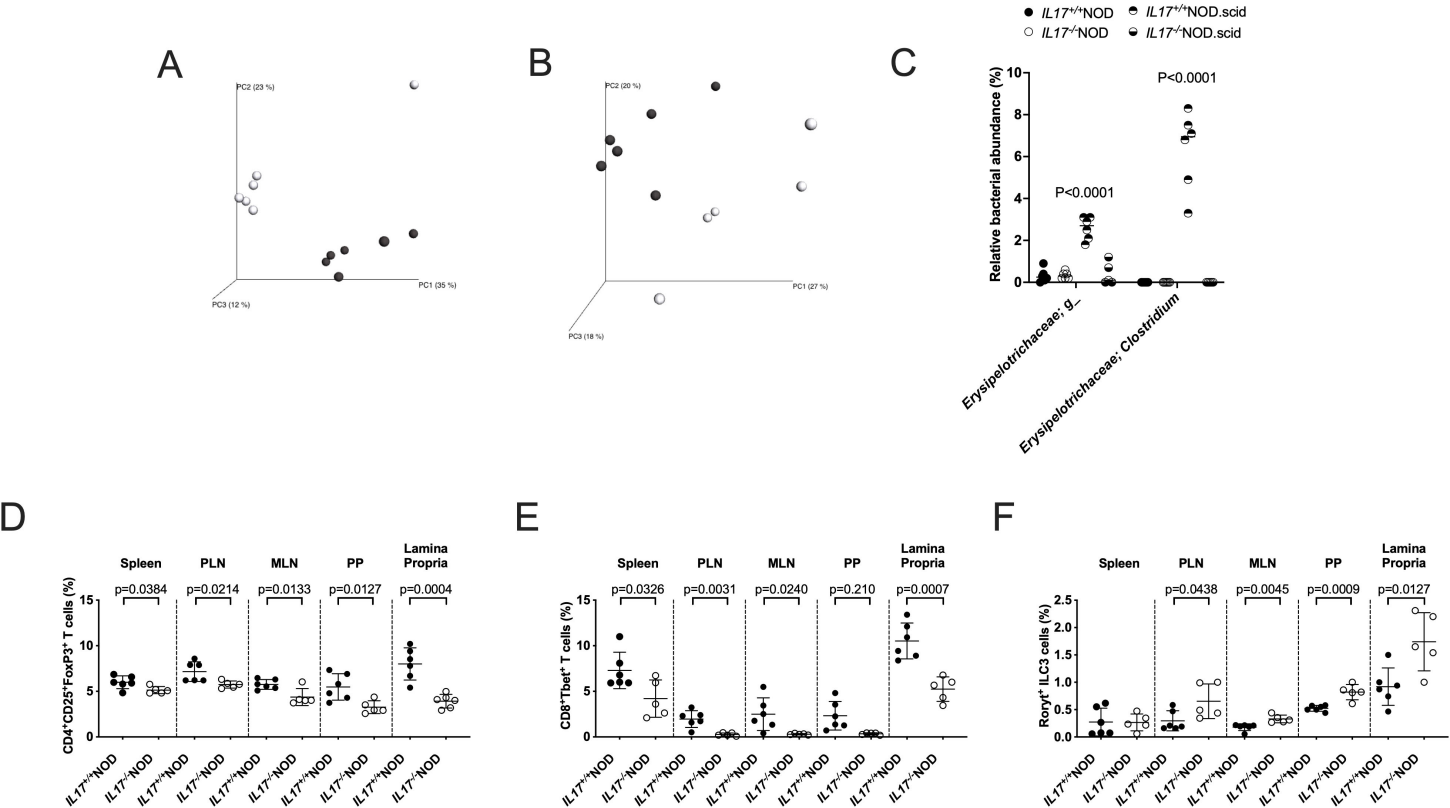
Faecal microbiota transplant recapitulate some IL-17-related immune changes. Principal co-ordinate analysis (PCA) plots of 16S rRNA sequencing from faecal microbiota of IL-17^+/+^ or IL-17^-/-^ NOD (**(A)** Black circles = IL-17^+/+^NOD, unfilled circles = IL-17^-/-^NOD) or NOD.scid mice (**(B)** Black circles = IL-17^+/+^NOD.scid, unfilled circles = IL-17^-/-^NOD.scid). **(C)** Bacterial abundances significantly altered by IL-17 gene deficiency determined by multiple T-tests. **(D–F)** IL-17^+/+^ or IL-17^-/-^ NOD faecal microbiota transplanted germ-free IL-17^+/+^ NOD mice were assessed two-weeks post-transfer for Tregs **(D)**, T-bet^+^CD8^+^ T cells **(E)** and RORγt^+^ ILC3s (all gated as in **Figure 2**). Data were assessed using a Student’s T-test. Lines represent mean + SD. Each data point represents an individual sample/mouse (n=5-6).

Whilst we found no difference in alpha diversity, a measure of the number of bacterial species (**Supplementary Figure S4A**), we observed significant differences in the beta diversity, a measure of bacterial composition (**Figure 4A**), between IL-17-sufficient and -deficient mice. This was also the case for the IL-17-deficient and -sufficient NOD.scid mice (**Figure 4B**; **Supplementary Figure S4B**). Further examination of the gut microbiota composition revealed no significantly altered bacteria at the genus level in immune-sufficient mice regardless of whether these were IL-17-sufficient or -deficient. However, two notable differences were observed in immune-deficient NOD.scid mice, where expansions were seen in the abundances within Erysipelotrichaceae - an unknown genus and Clostridia (**Figure 4C**). Interestingly, we observed no changes in the abundance of segmented filamentous bacteria, SFB, (**Supplementary Figure S4C**), which has been shown to induce Th17 T cells and associated with prevention of type 1 diabetes (29, 30). To fully interpret whether the changes in microbial beta diversity as a whole were responsible for the induction of the immune changes observed (**Figure 2**), we performed fecal microbiota transplant experiments, in which donor feces from IL-17-sufficient or -deficient NOD mice were orally gavaged into IL-17-sufficient germ-free NOD mice. Two-weeks post-gavage, mice were sacrificed and studied for immune changes. Here, we found that donors receiving IL-17-deficient fecal microbiota exhibited both reduced Treg and T-bet^+^CD8^+^ T cell proportions in all tissues studied, while ILC3s were increased in proportion in most tissues, compared to recipients of IL-17-sufficient fecal microbiota (**Figures 4D–F**). This suggested that the altered immune cell phenotype was linked to intestinal microbiota and could explain, in part, some of the differences we observed in the IL-17-deficient mice (**Figure 2**); however, not all the differences in immune cell phenotypes we found previously could be recapitulated in the colonized germ-free mice, including in Rorγt^+^ T cells (**Supplementary Figure S5**).

### IL-17 deficiency modulates intestinal homeostasis

**Figure 5:**
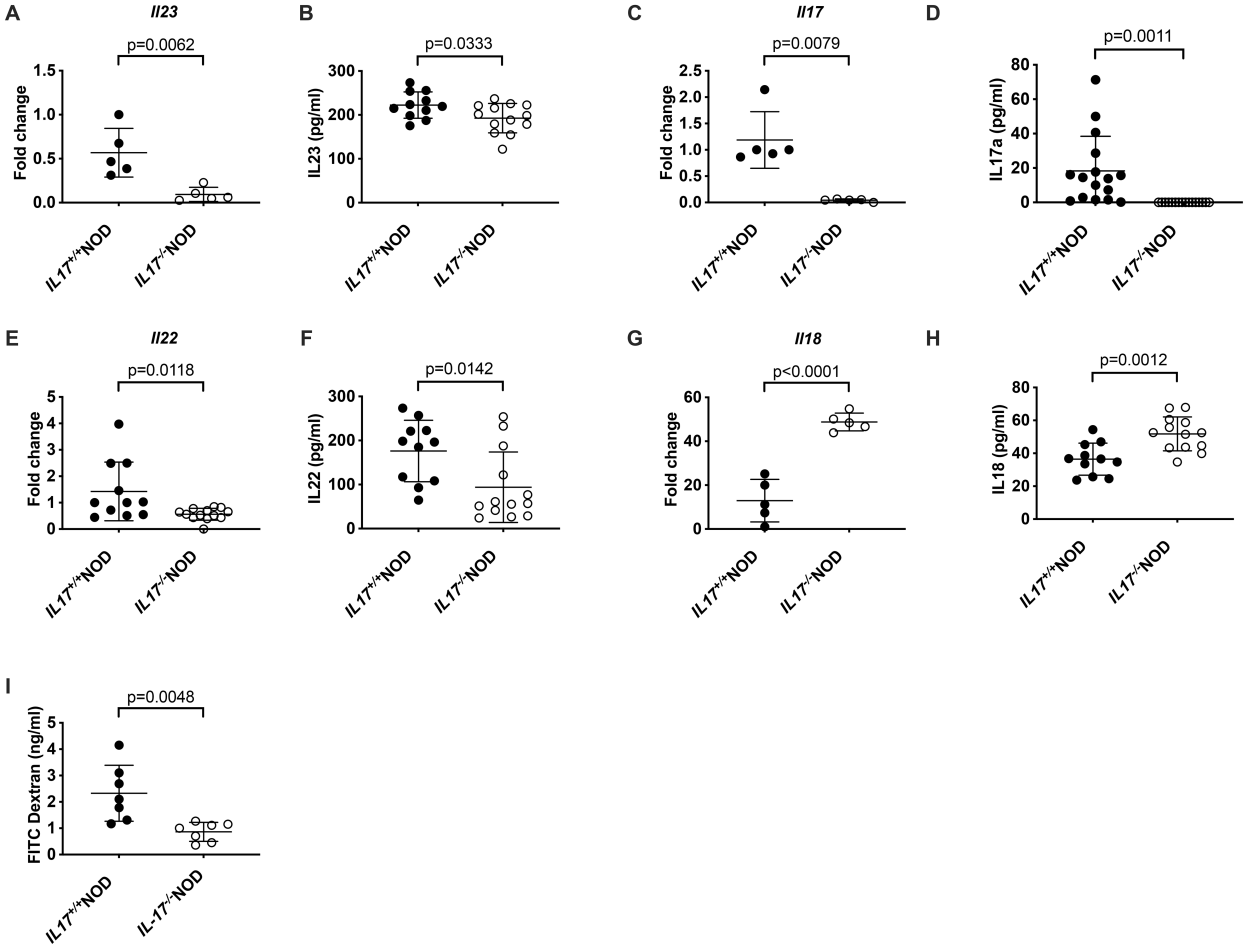
Intestinal changes in IL-17-deficient NOD mice. Small intestinal flush from 6-week-old IL-17^+/+^ or IL-17^-/-^NOD mice and tissue (distal small intestine) was collected for analysis of cytokines or gene expression. Gene expression was evaluated by qPCR for *Il-23***(A)**, *Il-17***(C)**, *Il-22***(E)** or *Il-18***(G)**, while protein expression of IL-23 **(B)**, IL-17A **(D)**, IL-22 **(F)** and IL-18 **(H)** was tested by ELISA. n=5-13, where n=5 is shown from one representative experiment, while n=11–13 is from two pooled experiments. **(I)***In vivo* assessment of intestinal permeability using orally-administered FITC-dextran (n=7; pooled from two independent experiments). Data were assessed for significance using either a Student’s T-test or Mann-Whitney test depending on if the data were normally distributed or not. Lines represent mean + SD.

As fecal microbiota composition was altered and directly responsible for some of the immune cell changes observed in IL-17-deficient NOD mice, we asked whether other intestinal factors, in addition to the gut microbiota, contributed to the changes in immune phenotype we had identified. IL-23, is a key cytokine involved in promoting both IL-17 and IL-22 secretion, which in turn induces IL-18, all of which help maintain the intestinal epithelial barrier and homeostasis. We found that IL-23 and IL-22 within the small intestine, at both the gene and protein level, were significantly reduced, while IL-18 was increased in IL-17-deficient mice compared to IL-17-sufficient mice (**Figures 5A–H**); however, no changes were found in either TGFβ or IFNγ within the small intestine (**Supplementary Figure S6A, B**). Gene expression analysis of antimicrobial peptides revealed that they too were reduced in the small intestine of IL-17-deficient mice (**Supplementary Figures S6C–H**). To determine whether IL-17-deficiency impacted intestinal permeability, we performed an *in vivo* FITC-dextran intestinal permeability assay and identified that IL-17-deficient mice had a less permeable gut, which was also evidenced by increased gene expression of both *claudin2* and *zonulin 1* (**Figure 5I**; **Supplementary Figures S6I, J**). This suggested that IL-17-deficiency promoted a stronger, less permeable intestinal barrier, largely through increased IL-18, which was associated with reduced anti-microbial responses.

### IL-17 deficiency modulates the intestinal epithelial barrier and prevents antigen presentation to autoreactive T cells

**Figure 6:**
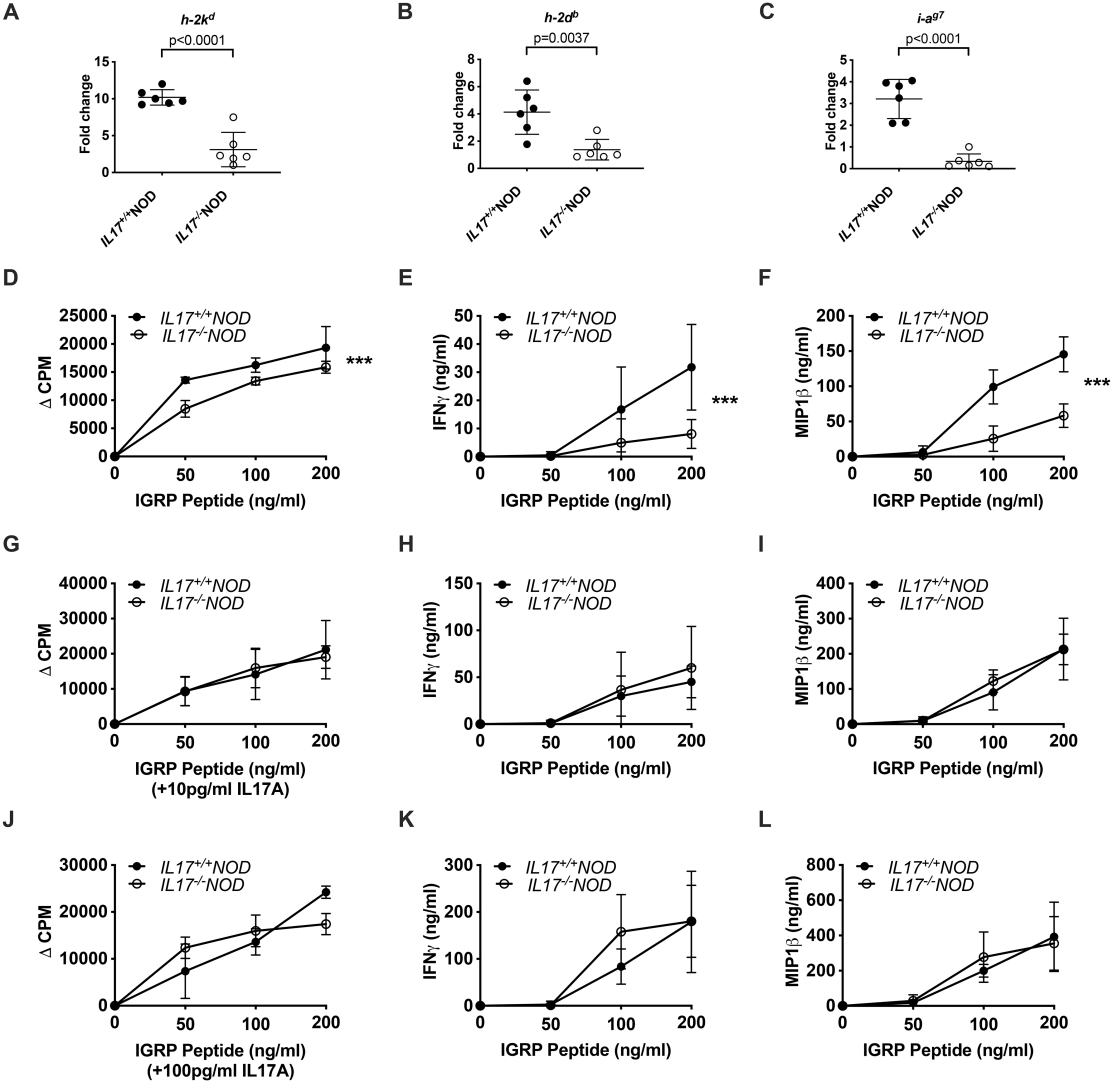
IL-17A rescues IL-17-deficient intestinal epithelial cell autoantigen presentation to autoreactive CD8^+^ T cells. Small intestinal flush from 6-week-old IL-17^+/+^ or IL-17^-/-^NOD mice and tissue (distal small intestine) was collected for analysis of gene expression, by qPCR, for MHC-I (*h-2^kd^***(A)** and *h-2^db^***(B)**) and MHC-II (*i-ag7***(C)**). Data were assessed for significance using either a Student’s T-test or Mann-Whitney test depending on whether the data were normally distributed or not. Lines represent mean + SD. Mitomycin-c-treated cecal epithelial cells from IL-17^+/+^NOD or IL-17^-/-^NOD were co-cultured 1:1 with NY8.3 CD8^+^ T cells in the presence or absence of IGRP_206–214_ peptide for 48 hours prior to supernatant removal for cytokine analysis and addition of ^3^H-thymidine for a further 18 hours to assess T cell proliferation. Data are shown from co-cultures without exogenous IL-17A **(D–F)**, or with 10pg/ml IL-17A **(G–I)** or 100pg/ml IL-17A **(J–L)**. In each experiment, triplicate wells were tested at each concentration of IGRP_206–214_ peptide, with data pooled from three independent experiments. Data were assessed for significance using a Two-way ANOVA.

Given that increased intestinal permeability has been associated with type 1 diabetes development (36), this could help explain why the IL-17-deficient mice, which have reduced intestinal permeability, are protected from the development of Type 1 diabetes. As autoreactive T cells can be activated in the intestine, prior to migration to the pancreas-draining lymph nodes (37–39), we asked whether intestinal epithelial cells were capable of presenting autoantigen differently to islet-specific T cells depending on whether they were from IL-17-deficient or -sufficient donors. To support this hypothesis, we observed reduced MHC-I and MHC-II expression in the intestine of IL-17-deficient NOD mice, compared to IL-17-sufficient NOD mice (**Figures 6A–C**). Since we observed IL-17-related changes predominantly in T-bet^+^CD8^+^ T cells (**Figures 2**, **4**), we assessed antigen presentation by intestinal epithelial cells to autoreactive IGRP_206-214_-reactive NY8.3 CD8^+^ T cells, co-culturing them with intestinal epithelial cells from IL-17-sufficient or -deficient NOD mice in the presence/absence of IGRP_206–214_ peptide. We found that IL-17-deficient epithelial cells were less able to present antigen to NY8.3^+^ CD8^+^ T cells, resulting in lower secreted concentrations of IFNγ and MIP-1β (**Figures 6D–F**). Interestingly, supplementing the media with low (10pg/ml) or high (100pg/ml) levels of IL-17A abolished the differences in antigen presentation and cytokine secretion (**Figures 6G–L**), suggesting that IL-17-deficiency was responsible for changes in antigen presentation but could be reversed by the presence of IL-17. This agrees with our *in vivo* data in **Figure 2**, whereby delays in diabetes were only seen when both host and immune cells lacked IL-17.

Together, our data indicate that IL-17-deficient intestinal epithelial cells, and interactions with the microbiome, are responsible for the changes in diabetes incidence observed through reduction of autoantigen-specific T cell activation within the intestine.

## Discussion

IL-17 is a key cytokine required for maintaining intestinal barrier functions and homeostasis including regulating commensal microbes. Our data show that IL-17-deficiency protects NOD mice from the development of Type 1 diabetes through contribution of both immune and non-immune cells.

From the immune cell perspective, we observed reduced Tregs and inflammatory T-bet^+^CD8^+^ T cells as well as changes to T cell activation, all of which would suggest reduced immune responses. While this reduction in Tregs may be surprising given we have improved protection from diabetes in IL-17-deficient mice, Tregs often require inflammatory responses to become activated, and thus due to the reduced inflammatory responses and T cell activation seen in IL-17-deficient mice, this may explain why Tregs were reduced. Interestingly, we observed no changes in Rorγt^+^ T cells, which are the main producers of IL-17. This is interesting as even in humans living with type 1 diabetes who received Ustekinumab (an anti-IL12/23 monoclonal antibody), there were no changes in Th17 cells in the peripheral blood until 1 year later (13). This suggests either some potential redundancy for Th17 cells, as other data in NOD mice have suggested (27), or that the Rorγt^+^ T cells express other cytokines like IFNγ, IL-2 and GMCSF, as evaluated by single cell sequencing in the Ustekinumab study (13).

Previous data in NOD mice have suggested that targeting IL-17 through gene silencing (shRNA knock down) in NOD zygotes did not impact diabetes development (27); however, our study suggests that genetic deletion of IL-17 results in protection from the development of Type 1 diabetes. It is possible that while IL-17 gene silencing reduced the level of IL-17A, it did not completely prevent expression of IL-17, and IL-17-secreting T cells could still be generated, albeit secreting lower levels of IL-17 than wild-type NOD mice. Thus, low levels of IL-17 may have been sufficient to maintain normal IL-17-related functionality in these mice. Furthermore, NOD mice treated with anti-IL17A antibodies at a young age (5 weeks of age) did not protect against diabetes development; however, mice were more protected when the therapy was administered at 10-weeks of age (28). The difference observed between our current study and the antibody treatment study could be that there was limited impact of the antibody therapy on IL-17-secreting T cells in the intestine or other mucosal tissues where they predominantly reside, which was not evaluated. Furthermore, it suggests that IL-17-secreting T cells can promote the development of Type 1 diabetes in mice with increased islet autoimmunity prior to diabetes development. Our data show that IL-17 addition to intestinal epithelial cell cultures with autoreactive CD8^+^ T cells can promote antigen presentation by the intestinal epithelial cells and proinflammatory cytokine secretion from the T cells. Thus, it is plausible that IL-17 presence in mice with more advanced islet damage (i.e. 10-weeks old), could promote antigen-specific T cell activation through a similar mechanism and that neutralizing antibodies to IL-17 may help prevent this.

Our data revealed that gut microbiota could transfer some, but not all, of the immune phenotype to the germ-free recipient mice, suggesting that microbiome and other factors such as cellular intrinsic IL-17-secretion, can modulate cell phenotypes. This could explain why some of the immune cell changes that could not be recapitulated, which may relate to the presence of the non-immune cells secreting IL-17, as the germ-free NOD mice are IL-17 sufficient. A limitation of this study is that we do not know the impact of cell-specific IL-17 deficiency and thus which of the changes we have observed are directly or indirectly related to IL-17 absence. It would be informative to evaluate this in future studies, especially focusing on T cell intrinsic IL-17 secretion and non-immune cell IL-17 secretion from intestinal epithelial cells.

IL-17 contributes to the pathogenesis of various autoimmune diseases such as rheumatoid arthritis, multiple sclerosis and intestinal bowel disease (25, 26, 40). Our data, and work by others (13, 14, 28), suggest that IL-17 can have an additive impact on the development of Type 1 diabetes, despite it being classed as a Th1-related disease. One novel mechanism that we have identified relates to the increased ability to promote antigen presentation by intestinal epithelial cells. This may be an important site prior to the development of Type 1 diabetes, as there are key changes in microbial composition (1–5), changes in intestinal structure (7) and permeability (6). Furthermore, it is clear that microbial mimicry and/or changes in the gut microbiota composition activate autoreactive T cells (37, 41–44), which may result in these T cells trafficking to the pancreas, as other studies have suggested (37–39). Thus, IL-17 may contribute to this process directly through increasing antigen presentation of these microbe-related autoantigens.

While we have observed novel immune changes related to IL-17-deficiency, human studies would be needed to confirm the translational potential of this work, particularly in regard to understanding how IL-17 blockade could impact intestinal antigen presentation to autoreactive T cells.

In summary, our data show that IL-17-deficiency can reduce the development of Type 1 diabetes through alterations in immune:epithelial:microbiome interactions that reduce the ability of intestinal epithelial cells to present antigen to autoreactive T cells. Further understanding of how we can target IL-17-specifically, either within different cell types or to those cells less impacted by IL-17-targetting therapy may greatly enhance our ability to better prevent the development of Type 1 diabetes and protect the insulin-producing beta cells.

## Data Availability

The datasets presented in this study can be found in online repositories. The names of the repository/repositories and accession number(s) can be found below: https://www.ncbi.nlm.nih.gov/, PRJNA1298311.

## Supplementary material

Supplementary Figure 1 Non-significant T cell changes and gating. Single cell suspensions were generated from 6-week-old mice (both sexes combined; n=6) from the spleen, pancreas-draining lymph node (PLN), mesenteric lymph node (MLN), Peyer’s Patches (PP) or small intestinal Lamina Propria. T cells were gated from live single TCRβ^+^ cells prior to gating on CD4^+^ or CD8^+^ T cells. RORγt^+^ or T-bet^+^ T cells were gated from either CD4^+^ or CD8^+^ T cells (**(A, B)** respectively), or from CD4^+^ T cells **(C)**. Representative gating of T-bet^+^CD8^+^ T cells with isotype control **(D)**, CD4^+^ T cell gating of CD44 and CD62L in **(E)** and CD8^+^ T cell gating of CD44 and CD62L **(F)**. **(G)** Proportion of γδ T cells. Data were assessed for significance by either a Student’s T-test (if parametric) or a Mann-Whitney test (if non-parametric). Data were pooled from 2 independent experiments. Lines represent mean + SD.

Supplementary Figure 2 ILC3 gating and other non-significant immune cell populations. Single cell suspensions were generated from 6-week-old mice (both sexes combined; n=6) from the spleen, pancreas-draining lymph node (PLN), mesenteric lymph node (MLN), Peyer’s Patches (PP) or small intestinal Lamina Propria. **(A)** Representative flow cytometry gating of RORγt ILC3s with Fluorescence-minus-one (FMO) and isotype controls. **(B, C)** Proportions of CD11b^+^CD11c^-^**(B)** or CD11b^+^CD11c^+^**(C)** cells gated from live, single TCRβ^-^CD19^-^ cells. **(D)** Proportions of CD19^+^ B cells gated from live, single TCRβ^-^ cells. Data were assessed for significance by either a Student’s T-test (if normally distributed) or a Mann-Whitney test (if non-normally distributed). Data were pooled from 2 independent experiments. Lines represent mean + SD.

Supplementary Figure 3 ILC1 and ILC2 data. Single cell suspensions were generated from 6-week-old mice (both sexes combined; n=6) from the spleen, pancreas-draining lymph node (PLN), mesenteric lymph node (MLN), Peyer’s Patches (PP) or small intestinal Lamina Propria. ILCs were gated from live, single TCRβ^-^TCRγδ^-^T-bet^+^ cells (ILC1; **(A)**) or TCRβ^-^TCRγδ^-^GATA-3^+^ cells (ILC2; **(B)**). Data were assessed for significance by either a Student’s T-test (if normally distributed) or a Mann-Whitney test (if non-normally distributed). Data were pooled from 2 independent experiments. Lines represent mean + SD.

Supplementary Figure 4 Faecal microbiota alpha diversity and SFB qPCR. Chao Average (alpha diversity measure) from 16S rRNA sequencing from faecal microbiota of IL-17^+/+^ or IL-17^-/-^ NOD (**(A)**; n=6) or IL-17^+/+^ or IL-17^-/-^ NOD.scid (**(B)**; n=6). **(C)** Segmented filamentous bacteria (SFB) was assessed by qPCR from faecal microbiota samples in A. Data were assessed for significance by a Student’s T-test. Lines represent mean + SD.

Supplementary Figure 5 Non-significant immune changes post-faecal microbiota transplantation into germ-free NOD mice. IL-17^+/+^ or IL-17^-/-^ NOD faecal microbiota transplanted germ-free IL-17^+/+^ NOD mice were assessed two-weeks post-transfer for immune cell proportions. Cell proportions investigated include CD4^+^ T cells **(A)**, CD8^+^ T cells **(B)**, CD4^+^ and CD8^+^ T cell activation assessed using CD44 and CD62L **(C–H)**, CD4^+^ and CD8^+^ RORγt^+^ T cells **(I, J)** and T-bet^+^CD4^+^ T cells **(K)**. Cells were gated as outlined in **Figure 2**/**Supplementary Figure S1**. Data were assessed for significance by either a Student’s T-test (if parametric) or a Mann-Whitney test (if non-parametric). Each data point represents an individual sample/mouse (n=6; pooled from two experiments). Lines represent mean + SD.

Supplementary Figure 6 Intestinal cytokine, anti-microbial peptide and permeability markers. Small intestinal flush from 6-week-old IL-17^+/+^ or IL-17^-/-^NOD mice and tissue (distal small intestine) was collected for analysis of cytokines or gene expression. **(A, B)** IFNγ and TGFβ1 were measured from the small intestinal flush by ELISA. Gene expression was evaluated by qPCR for anti-microbial peptides **(C–H)** or intestinal permeability markers **(I, J)**. n=5-13, where n=5 is shown from one representative experiment, while n=11–13 is from two pooled experiments. Data were assessed for significance using either a Student’s T-test or Mann-Whitney test depending on if the data were normally distributed or not. Lines represent mean + SD.
