# Supplementary material for: IL17-deficient NOD mice are protected from autoimmune diabetes due to decreased antigen presentation and T cell activation
Source: Front Immunol. 2026 Jan 12;16:1728313. doi: 10.3389/fimmu.2025.1728313 (PMC12850519; doi:10.3389/fimmu.2025.1728313)
Supplement: Supplementary Figure 1 — Non-significant T cell changes and gating. Single cell suspensions were generated from 6-week-old mice (both sexes combined; n=6) from the spleen, pancreas-draining lymph node (PLN), mesenteric lymph node (MLN), Peyer’s Patches (PP) or small intestinal Lamina Propria. T cells were gated from live single TCRβ+ cells prior to gating on CD4+ or CD8+ T cells. RORγt+ or T-bet+ T cells were gated from either CD4+ or CD8+ T cells ((A, B) respectively), or from CD4+ T cells (C). Representative gating of T-bet+CD8+ T cells with isotype control (D), CD4+ T cell gating of CD44 and CD62L in (E) and CD8+ T cell gating of CD44 and CD62L (F). (G) Proportion of γδ T cells. Data were assessed for significance by either a Student’s T-test (if parametric) or a Mann-Whitney test (if non-parametric). Data were pooled from 2 independent experiments. Lines represent mean + SD. [file Presentation1.pptx]

## Slide 1
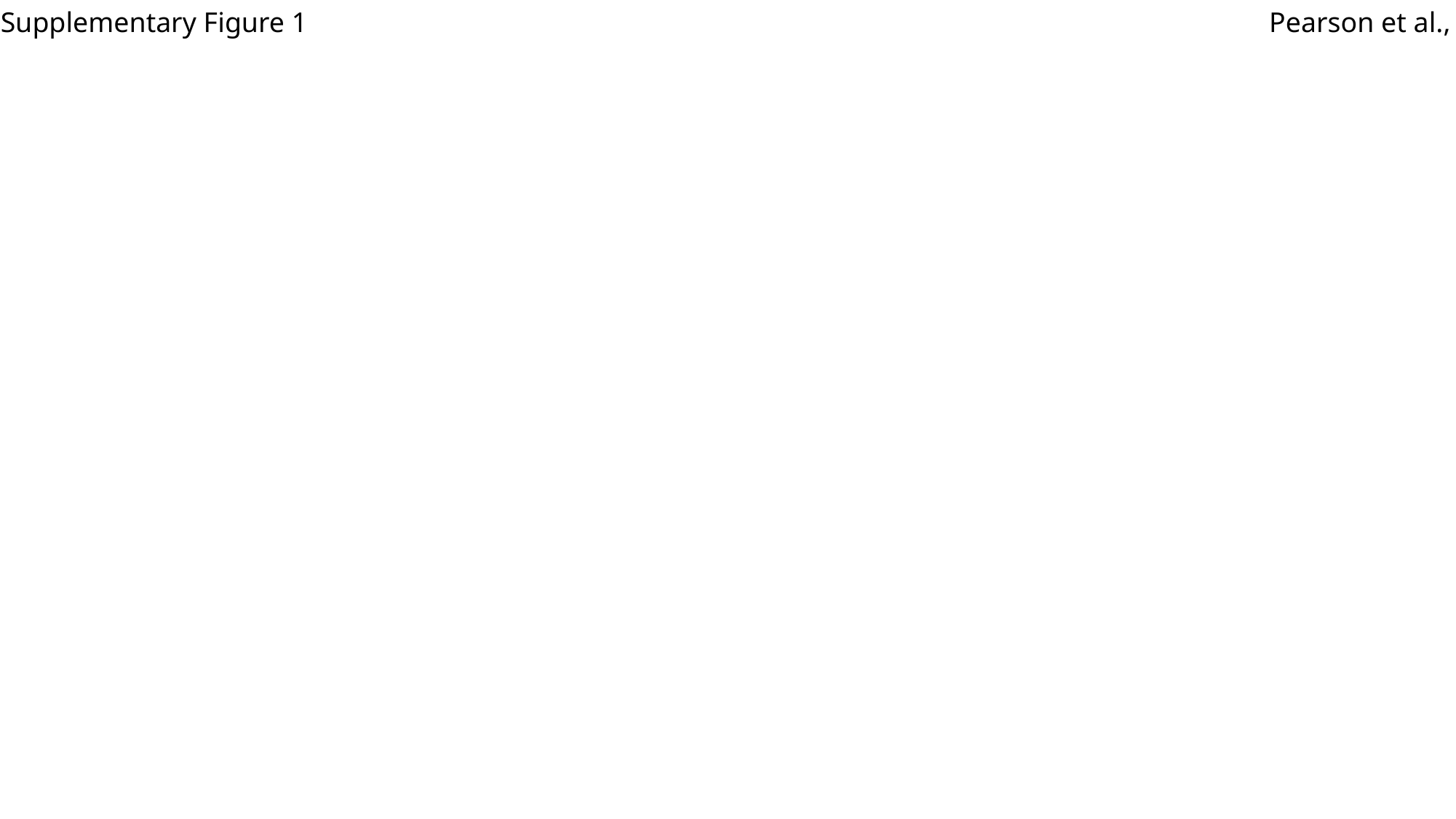

Supplementary Figure 1
Pearson et al.,

## Slide 2
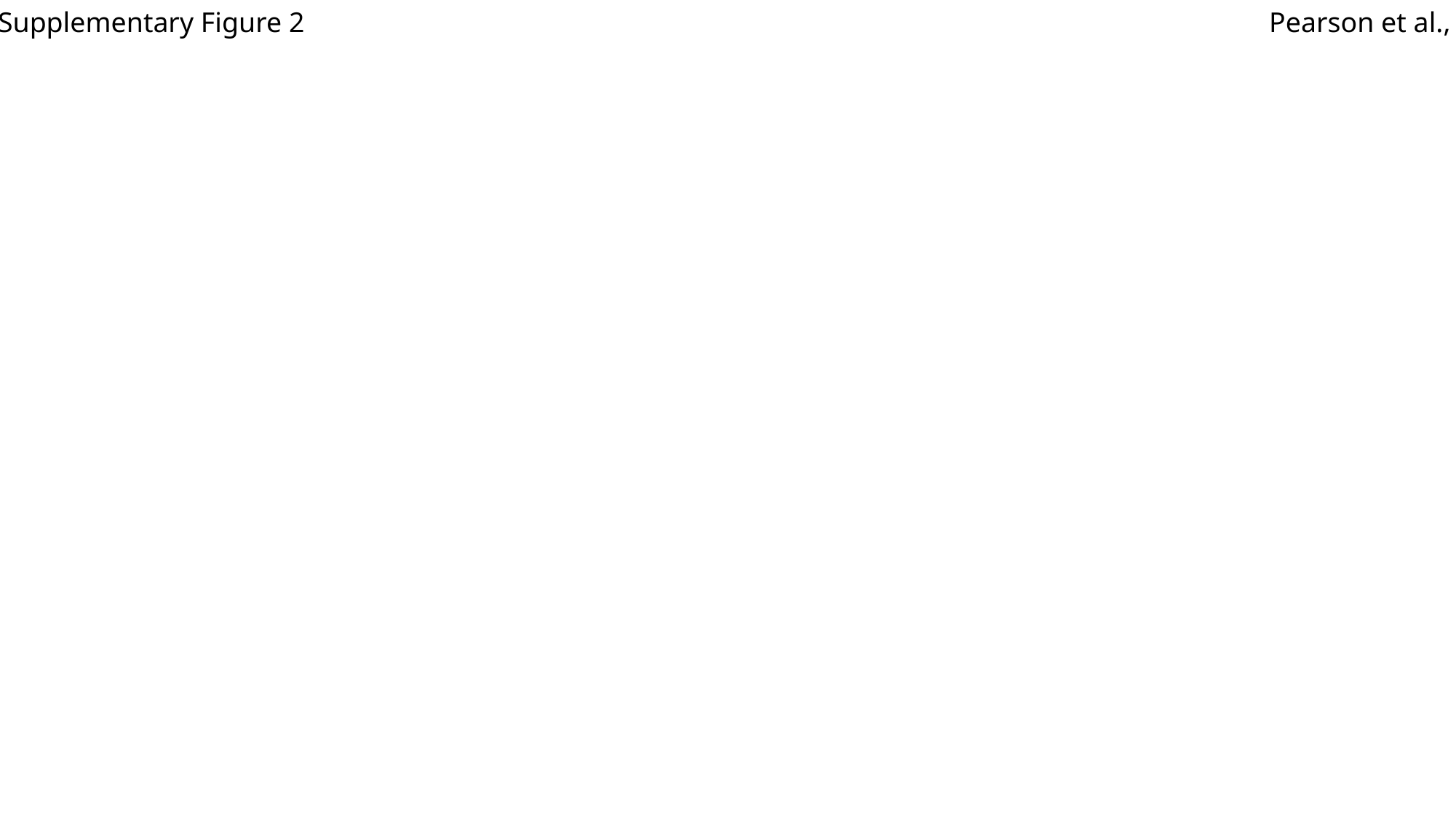

Supplementary Figure 2
Pearson et al.,

## Slide 3
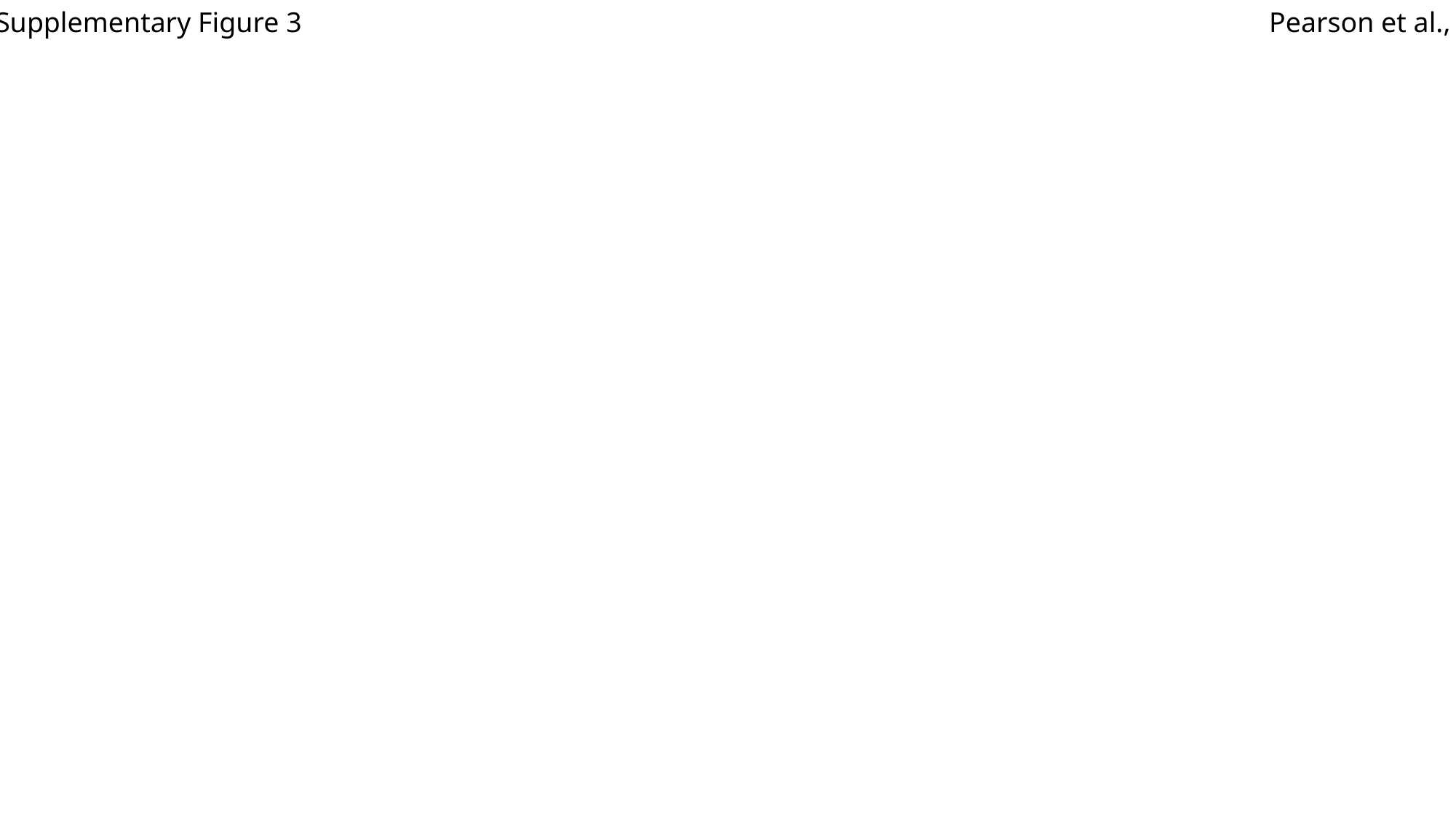

Supplementary Figure 3
Pearson et al.,

## Slide 4
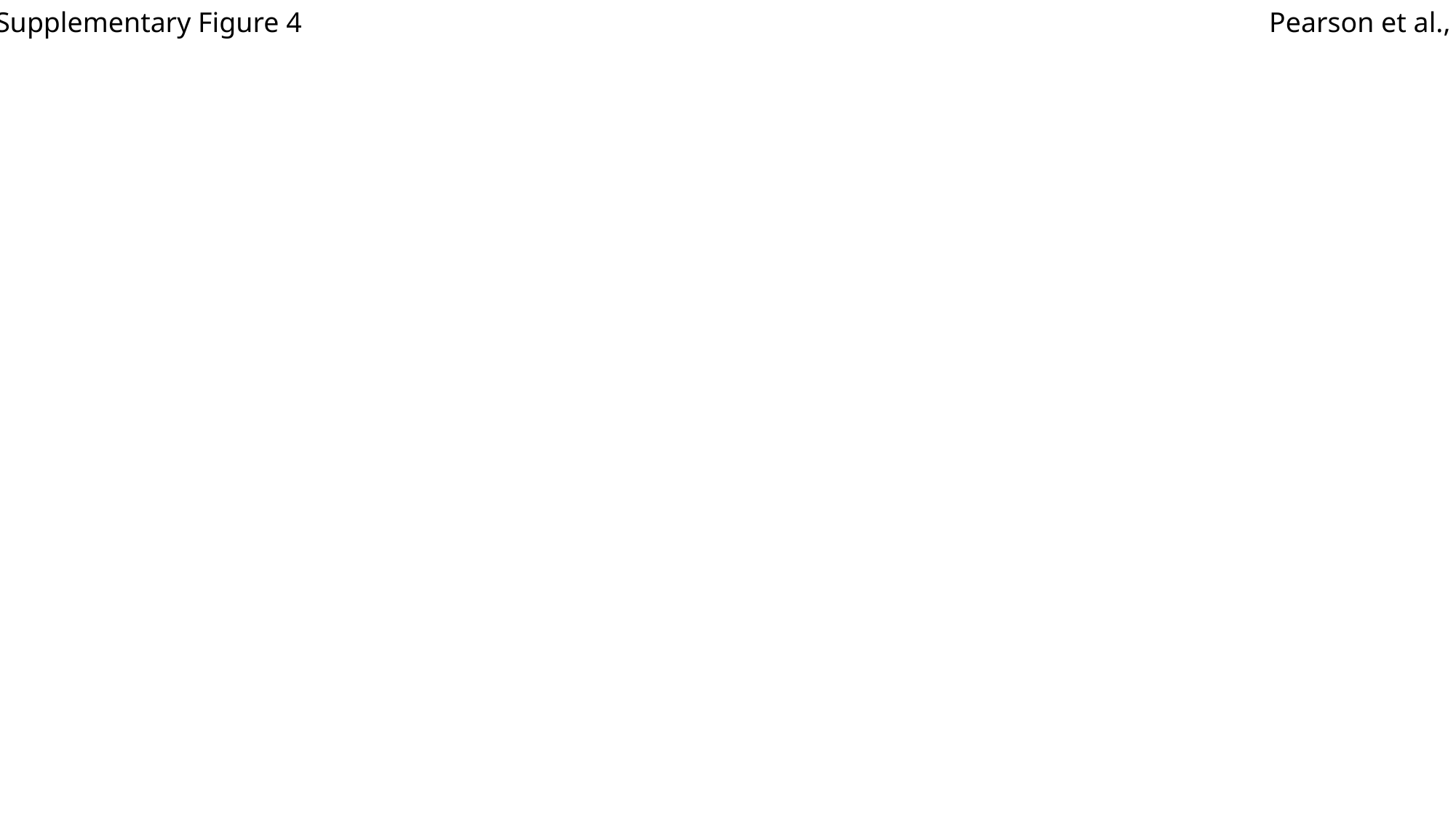

Supplementary Figure 4
Pearson et al.,

## Slide 5
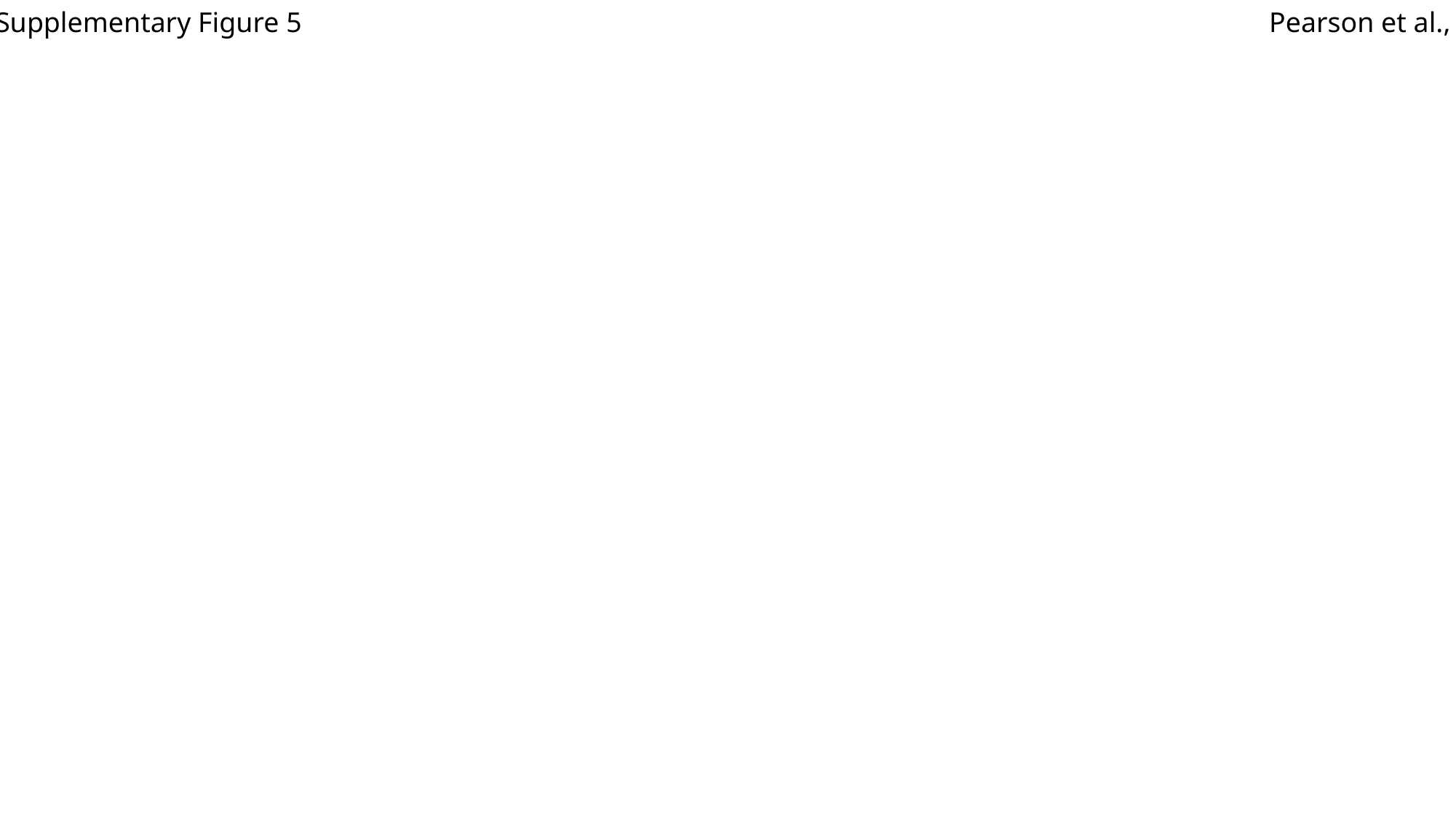

Supplementary Figure 5
Pearson et al.,

## Slide 6
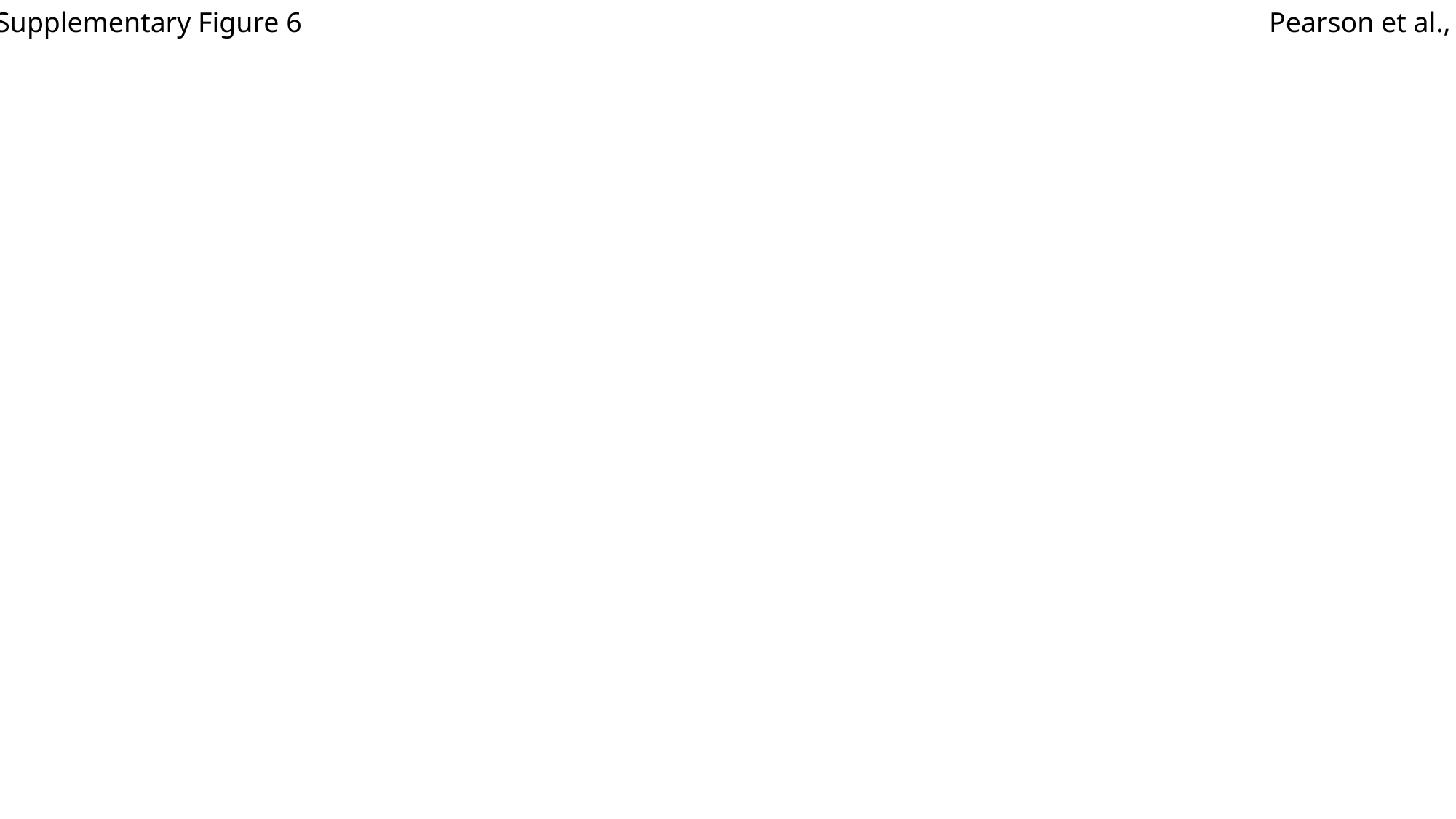

Supplementary Figure 6
Pearson et al.,

## Slide 7
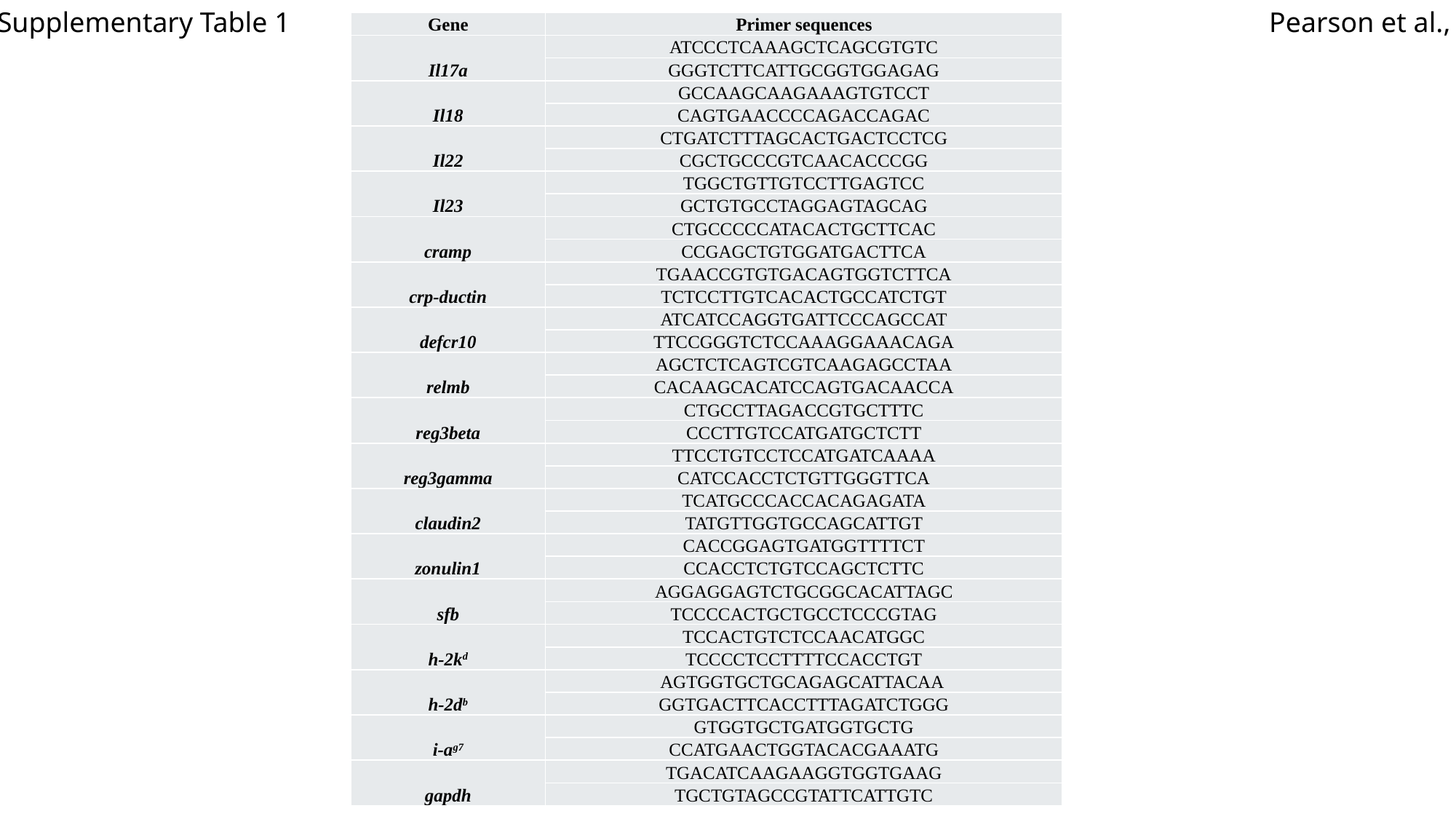

Supplementary Table 1
Pearson et al.,
| Gene | Primer sequences |
| --- | --- |
| Il17a | ATCCCTCAAAGCTCAGCGTGTC |
| | GGGTCTTCATTGCGGTGGAGAG |
| Il18 | GCCAAGCAAGAAAGTGTCCT |
| | CAGTGAACCCCAGACCAGAC |
| Il22 | CTGATCTTTAGCACTGACTCCTCG |
| | CGCTGCCCGTCAACACCCGG |
| Il23 | TGGCTGTTGTCCTTGAGTCC |
| | GCTGTGCCTAGGAGTAGCAG |
| cramp | CTGCCCCCATACACTGCTTCAC |
| | CCGAGCTGTGGATGACTTCA |
| crp-ductin | TGAACCGTGTGACAGTGGTCTTCA |
| | TCTCCTTGTCACACTGCCATCTGT |
| defcr10 | ATCATCCAGGTGATTCCCAGCCAT |
| | TTCCGGGTCTCCAAAGGAAACAGA |
| relmb | AGCTCTCAGTCGTCAAGAGCCTAA |
| | CACAAGCACATCCAGTGACAACCA |
| reg3beta | CTGCCTTAGACCGTGCTTTC |
| | CCCTTGTCCATGATGCTCTT |
| reg3gamma | TTCCTGTCCTCCATGATCAAAA |
| | CATCCACCTCTGTTGGGTTCA |
| claudin2 | TCATGCCCACCACAGAGATA |
| | TATGTTGGTGCCAGCATTGT |
| zonulin1 | CACCGGAGTGATGGTTTTCT |
| | CCACCTCTGTCCAGCTCTTC |
| sfb | AGGAGGAGTCTGCGGCACATTAGC |
| | TCCCCACTGCTGCCTCCCGTAG |
| h-2kd | TCCACTGTCTCCAACATGGC |
| | TCCCCTCCTTTTCCACCTGT |
| h-2db | AGTGGTGCTGCAGAGCATTACAA |
| | GGTGACTTCACCTTTAGATCTGGG |
| i-ag7 | GTGGTGCTGATGGTGCTG |
| | CCATGAACTGGTACACGAAATG |
| gapdh | TGACATCAAGAAGGTGGTGAAG |
| | TGCTGTAGCCGTATTCATTGTC |
